# Fecal Metaproteomic Analysis Reveals Unique Changes of the Gut Microbiome Functions After Consumption of Sourdough *Carasau* Bread

**DOI:** 10.3389/fmicb.2019.01733

**Published:** 2019-07-30

**Authors:** Marcello Abbondio, Antonio Palomba, Alessandro Tanca, Cristina Fraumene, Daniela Pagnozzi, Monica Serra, Fabio Marongiu, Ezio Laconi, Sergio Uzzau

**Affiliations:** ^1^Department of Biomedical Sciences, University of Sassari, Sassari, Italy; ^2^Porto Conte Ricerche, Science and Technology Park of Sardinia, Alghero, Italy; ^3^Department of Biomedical Sciences, University of Cagliari, Cagliari, Italy

**Keywords:** gut microbiota, metagenomics, metaproteomics, food processes, sourdough, diet

## Abstract

Sourdough-leavened bread (SB) is acknowledged for its great variety of valuable effects on consumer's metabolism and health, including a low glycemic index and a reduced content of the possible carcinogen acrylamide. Here, we aimed to investigate how these effects influence the gut microbiota composition and functions. Therefore, we subjected rats to a diet supplemented with SB, baker's yeast leavened bread (BB), or unsupplemented diet (chow), and, after 4 weeks of treatment, their gut microbiota was analyzed using a metaproteogenomic approach. As a result, diet supplementation with SB led to a reduction of specific members of the intestinal microbiota previously associated to low protein diets, namely *Alistipes* and *Mucispirillum*, or known as intestinal pathobionts, i.e., *Mycoplasma*. Concerning functions, asparaginases expressed by *Bacteroides* were observed as more abundant in SB-fed rats, leading to hypothesize that in their colonic microbiota the enzyme substrate, asparagine, was available in higher amounts than in BB- and chow-fed rats. Another group of protein families, expressed by *Clostridium*, was detected as more abundant in animal fed SB-supplemented diet. Of these, manganese catalase, small acid-soluble proteins (SASP), Ser/Thr kinase PrkA, and V-ATPase proteolipid subunit have been all reported to take part in *Clostridium* sporulation, strongly suggesting that the diet supplementation with SB might promote environmental conditions inducing metabolic dormancy of *Clostridium* spp. within the gut microbiota. In conclusion, our data describe the effects of SB consumption on the intestinal microbiota taxonomy and functions in rats. Moreover, our results suggest that a metaproteogenomic approach can provide evidence of the interplay between metabolites deriving from bread digestion and microbial metabolism.

## Introduction

Among bakery products, bread is the most abundantly consumed food worldwide, with an increase in demand for products containing wholegrain, high in fiber, or obtained through “health-promoting” processing, such as sourdough leavening. Use of sourdough has been shown to improve flavor, structure, and shelf life of baked bread, due to its differences in chemical and physical features compared to baker's yeast leavening (Gobbetti et al., 2016).

Further, cereal fermentations are widely recognized as of great potential in improving the nutritional quality of food ingredients and their healthy effects. A number of studies claimed that specific cereal matrix and/or the bakery processes used to produce bread might lead to the improvement of clinical parameters in habitual consumers (Korem et al., 2017). Sourdough leavening actively retards starch digestibility, leading to low glycemic responses, and may increase the production of non-digestible polysaccharides that escape the small intestine, together with grain fibers, eventually feeding the colonic microbiota (Maioli et al., 2008; Scazzina et al., 2009; Sanna et al., 2018). To this end, selected species of lactic acid bacteria (LAB) have been tested with the aim of improving bread quality (De Vuyst et al., 2014). Also, sourdough leavening modulates levels and bioaccessibility of bioactive compounds, and improves mineral bioavailability (Di Nunzio et al., 2018).

Different enzymatic activities in sourdough and baker's yeast fermentation might be responsible for specific hydrolysis of proteins and polysaccharides. Protein hydrolysis, in turn, may affect the absorption of bioactive compounds as well as other metabolites impacting on the host physiology. Sourdough has also been proposed to yield bread with highly degraded gluten that may be appropriate for gluten intolerant individuals (i.e., with non-celiac gluten sensitivity) (Gobbetti et al., 2018a,b).

Strikingly, sourdoughs fermentation has also been demonstrated to reduce the acrylamide content in wheat bread (Bartkiene et al., 2013). As for other food products, factors affecting acrylamide formation during bread production are acrylamide precursors (mainly asparagine), reducing sugars and specific processing conditions. These sourdough features have important practical implications, since acrylamide neurotoxicity, genotoxicity, carcinogenicity, and reproductive toxicity have been demonstrated (Keramat et al., 2011), and bakery products account for around 20% of human exposure to acrylamide.

Based on these premises, this study was designed to gain insights into the complex interplay between sourdough effects on bread preparation and the gut microbiota (GM) taxonomy and functional activities, and, in turn, to elucidate its possible impact on consumer's metabolism and health. To date, the specific contribution of sourdough bread consumption to the functional activities of microbiota has not been evaluated. Therefore, we compared the composition and the active functions of microbial intestinal communities in three groups of rats fed a diet supplemented with sourdough bread (SB), baker's yeast leavened bread (BB), or unsupplemented diet. Specifically, we choose to evaluate the impact of sourdough consumption in rats fed a calorie-restricted diet, based on low fat high fiber composition, to avoid GM modifications that have been already associated to high fat and/or high sugars obesogenic diets. With this approach, we also minimized the potential confounding effects due to individual difference in food intake, generally occurring in animals fed *ad libitum* (AL).

## Materials and Methods

### Animals and Samples

A total of 16 Fischer 344 rats (10 weeks old, male) were purchased from Charles River Laboratories Italia, SRL (Calco, Italy) together with the manufacturer's animal chow VRF1 (P) 811900 (4.5% of fat). Animals were distributed two per cage and maintained on daily cycles of alternating 12 h light-darkness (light on at 11 p.m., light off at 11 a.m.), with food and water available AL. Animal studies were reviewed and approved by the Institutional Animal Care and Use Committee of the University of Cagliari and were performed in accordance with the relevant guidelines and regulations (authorization of the Italian Health Ministry No. 840/2016-PR). After 2 weeks of acclimatization, rats were divided in four groups of four rats each and were exposed to the following feeding schedule. The first group was continued on AL chow diet (“chow-AL” group), while the other three groups were fed a calorie-restricted (CR) diet, calculated as 70% of AL food intake, as previously reported (Fraumene et al., 2018; Tanca et al., 2018). Among CR-fed rats, one group received only laboratory chow (“chow” group), while the remaining two groups were supplemented (15% w/w) with a typical Sardinian bread (*carasau* bread, produced by a local bakery company), leavened with BB (“BB” group) or SB (“SB” group), respectively.

Animals were weighed weekly and sacrificed after 4 weeks of treatment with their respective dietary regimens.

Glycemia was measured with Glucose Analyzer II (Beckman Coulter, Brea, CA, USA). Blood samples were taken from the tail vein 1 h prior to food delivery or 2 h after food delivery.

Stool, liver, and colonic content samples were collected from CR-fed rats after 4 weeks of diet treatment, whereas AL-fed rats were used merely as a growth control. Fecal samples were collected from all animals, apart from one rat belonging to the “chow” group. Colonic content and liver samples were collected from all animals after sacrifice. All samples were immediately stored at −80°C until use. At the time of the analyses, stool samples were thawed at 4°C and two portions were collected from each of them for protein and DNA extraction, respectively; colonic contents were directly processed for DNA extraction, whereas liver samples were directly processed for protein extraction.

### DNA Extraction and 16S rRNA Gene Sequencing

DNA was extracted from 11 fecal samples and 12 colon content samples, collected from rats belonging to “chow”, “BB”, and “SB” groups. Extraction was performed according to QIAamp Fast Stool Kit protocol (QIAGEN, Hilden, Germany). The extracted DNA was purified according to E.Z.N.A.^®^ Soil DNA Kit (Omega Bio-Tek, Norcross, GA, USA). DNA quality and yield were evaluated via agarose gel and Qubit fluorometer (Life Technologies, CA, USA). Libraries were constructed using Illumina's recommendations as implemented in 16S Metagenomic Sequencing Library Preparation guide. To amplify the variable region 4 of the 16S rRNA gene, we used the 515F and 806R primers (GTGCCAGCMGCCGCGGTAA and GGACTACHVGGGTWTCTAAT, respectively) modified to contain adaptors for MiSeq sequencing. Two separate gene amplification reactions were performed for each sample, pooled together and cleaned up using AMPure XP (Beckman Coulter) magnetic beads. The next PCR attached dual index barcodes using the Illumina Nextera XT kit so that the PCR products may be pooled and sequenced directly. The final quality control and quantification of the libraries were conducted using a Bioanalyzer 2100 (Agilent Technologies, Santa Clara, CA, USA). DNA sequencing was performed on the Illumina MiSeq platform, using v3 chemistry according to the manufacturer's specifications, to generate paired-end reads of 201 bases in length in each direction. Data quality control and analyses were performed using the QIIME pipeline (v.1.9.1) (Caporaso et al., 2010). The overlapping paired-end reads were merged using the script join_paired_ends.py inside the QIIME package. OTUs generation was done using a pipeline based on USEARCH's OTU clustering recommendations (http://www.drive5.com/usearch/manual/otu_clustering.html) using the closed-reference OTU picking to allow clustering of 16S rRNA gene sequences, as previously described (Tanca et al., 2017). Reads were clustered at 97% identity using UCLUST to produce OTUs (Edgar, 2010). Taxonomy was then assigned using the Greengenes 13_8 database (DeSantis et al., 2006).

### Protein Extraction and Proteomic Analysis

Eleven fecal samples and 12 liver samples, collected from rats belonging to “chow,” “BB,” and “SB” groups, were subjected to bead-beating and heating/freezing steps after resuspension in an SDS-based reducing extraction buffer, as described earlier (Tanca et al., 2014). Protein extracts were cleaned up, alkylated, and trypsin digested according to the filter-aided sample preparation procedure (Wisniewski et al., 2009), with minor modifications illustrated elsewhere (Tanca et al., 2013, 2015).

Liquid chromatography (LC)-tandem mass spectrometry (MS/MS) analyses were performed on an LTQ Orbitrap Velos mass spectrometer (Thermo Fisher Scientific, Waltham, MA, USA), operating with an EASY-spray source, interfaced with an UltiMate 3000 RSLCnano LC system (Thermo Fisher Scientific). Samples were run in a randomized order. After loading, peptide mixtures (4 μg per run) were loaded, concentrated, and desalted on a trapping pre-column (Acclaim PepMap C18, 75 μm × 2 cm nanoViper, 3 μm, 100 Å, Thermo Fisher Scientific), using 0.2% formic acid at a flow rate of 5 μl/min. The peptide separation was performed with a C18 EASY-spray column (PepMap RSLC C18, 75 μm × 50 cm, 2 μm, 100 Å, Thermo Fisher Scientific) at 35°C with a flow rate of 250 nL/min for 247 min, using the following two-step gradient of eluent B (0.2% formic acid in 95% ACN) in eluent A (0.2% formic acid in 5% ACN): 2.5–37.5% for 242 min and 37.5–99% for 5 min.

The mass spectrometer was set up in a data dependent MS/MS mode, where a full scan spectrum (from 375 to 2,000 m/z) is followed by MS/MS spectra, under direct control of the Xcalibur software. The instrument operated in positive mode. The temperature of ion transfer capillary and the spray voltage were set to 250°C and 1.85 kV, respectively. Full scans and MS/MS spectra were acquired in the Orbitrap with resolutions of 60,000 and 7,500 at 400 m/z, respectively. The automatic gain control was set to 1,000,000 ions, and the lock mass option enabled on a protonated polydimethylcyclosiloxane background ion as internal recalibration for accurate mass measurements (Olsen et al., 2005). Peptide ions were selected as the 10 most intense peaks of the previous scan; the signal threshold for triggering an MS/MS event was set to 500 counts, and dynamic exclusion was set to 30 s. Higher-energy collisional dissociation was used as the fragmentation method, by applying a 35% value for normalized collision energy, an isolation width of m/z 3.0, a Q-value of 0.25, and an activation time of 0.1 ms. Nitrogen was used as the collision gas.

Microbial peptide identification was carried out using the Proteome Discoverer informatic platform (version 2.0; Thermo Fisher Scientific), with Sequest-HT as search engine and Percolator for peptide validation (FDR < 1%). Search parameters were set as follows: precursor mass threshold 350–5,000 Da; minimum peak count 6; signal-to-noise threshold 2; enzyme trypsin; maximum missed cleavage sites 2; peptide length range 5–50 amino acids; precursor mass tolerance 10 ppm; fragment mass tolerance 0.02 Da; dynamic modification methionine oxidation; static modification cysteine carbamidomethylation. Two parallel processing nodes were used. The first processing node was built on a combination of three microbial sequence databases: (i) a collection of metagenomic sequences obtained in house from rat fecal samples (11,510,359 sequences in total) and processed according to previous reports (Tanca et al., 2016); (ii) a publicly available mouse metagenomic dataset (ftp://penguin.genomics.cn/pub/10.5524/100001_101000/100114/Genecatalog/184sample_2.6M.GeneSet.pep.gz) (Xiao et al., 2015) merged with a collection of metagenomic sequences obtained in house from mouse fecal samples (9,825,357 sequences in total); (iii) a pseudometagenome comprising all UniProtKB (release 2017_11) sequences belonging to the 54 microbial genera (NCBI taxonomy IDs: 157, 270, 286, 434, 469, 475, 816, 838, 841, 872, 970, 1253, 1263, 1279, 1301, 1386, 1485, 1578, 1654, 1678, 1716, 1883, 2093, 2152, 29407, 33024, 33042, 33926, 35832, 40544, 51514, 82373, 86331, 119852, 121871, 129337, 150247, 174708, 189330, 191303, 207244, 239759, 239934, 248038, 283168, 346096, 375288, 416916, 447020, 497726, 572511, 574697, 577309, 869896) detected in the 16S rRNA analysis described in this study with an abundance > 0.1% in at least one sample (17,389,183 sequences in total). All microbial sequence databases have been deposited in PRIDE along with MS data. The second processing node was built on a database containing the protein sequences belonging to the order Rodentia and deposited in UniProtKB/SwissProt (release 2017_11; 26,656 sequences in total). Liver samples were subjected to the second processing node only.

Taxonomic and functional annotation was performed using multiple strategies. MEGAN v.6.8.19 was used as first annotation option (Huson et al., 2016). Protein sequences were preliminary subjected to a DIAMOND (v.0.8.22) search against the NCBI-nr database (2017/09 update), using the blastp command with default parameters (Buchfink et al., 2015); then, DIAMOND outputs were loaded on MEGAN and lowest common ancestor (LCA) classification was performed using default parameters. Furthermore, the Unipept web application (v.3.3.4; https://unipept.ugent.be) was used to carry out an LCA classification of the identified peptide sequences (Mesuere et al., 2017). Functional annotation was accomplished by aligning the identified protein sequences against a database containing all bacterial sequences from UniProtKB/Swiss-Prot (release 2017_09) using DIAMOND (blastp module, e-value threshold 10^−5^); UniProtKB/Swiss-Prot accession numbers were subsequently exploited to retrieve protein family information from the UniProt website via the “retrieve” tool (Pundir et al., 2016). Metaproteomic spectral count data obtained for each sample were aggregated based on the functional and taxonomic annotation levels, generating abundance tables of family-specific and genus-specific protein families.

### Statistical Analysis and Graph Generation

Differential analysis was performed on read (16S rRNA gene sequencing) and spectral (metaproteomics) count data using the edgeR package available in a Galaxy server (https://bioinf-galaxian.erasmusmc.nl/galaxy) (Robinson et al., 2010). The *p*-value lists provided by edgeR were subsequently subjected to a multiple testing adjustment based on a sequential goodness of fit (SGoF) metatest (Carvajal-Rodriguez et al., 2009) using the SGoF+ software (v.3.8) with default parameters (Carvajal-Rodriguez and de Una-Alvarez, 2011). An adjusted *p*-value < 0.05 was considered as the threshold for statistical significance of differential results. Features with missing value(s) in more than one group were filtered out. Beta diversity among groups was inspected by performing principal coordinate analysis (PCoA) and permutational multivariate analysis of variance (PERMANOVA) on taxonomic data at the genus level, using the web application MicrobiomeAnalyst (http://www.microbiomeanalyst.ca) (Dhariwal et al., 2017). In addition, using GraphPad Prism (v.5.03), we performed one-way analysis of variance (ANOVA) following by Bonferroni comparison on all pairs of groups (alpha-value = 0.05) on body weight and glycemia data, and Kruskal-Wallis test followed by Dunn's multiple comparison test (alpha-value = 0.05) on alpha-diversity data (Simpson and Shannon indexes), in order to evaluate the significance of variation among groups.

Heatmaps were generated starting from relative abundance data using the web application Morpheus (https://software.broadinstitute.org/morpheus), while line graphs were generated with GraphPad Prism.

## Results

### Experimental Design and General Metrics

In this study we investigated the effect of chow diet supplementation with Sardinian typical *carasau* bread leavened with standard baker's yeast or sourdough on the GM of young rats. To this end, animals were fed under a CR regimen with three different diets, namely, chow only, chow plus BB or chow plus SB; in addition, a fourth control group (“chow-AL”) was fed AL.

Animal weights were recorded weekly during the dietary treatment. As expected, a significant difference was observed starting from the first week between the “chow-AL” control group and the three CR-fed groups (one-way ANOVA plus Bonferroni's comparison for multiple testing, Supplementary Figure 1).

Glycemia was also evaluated in each group after 1, 3, and 4 weeks, and values were measured both 1 h before and 2 h after the meal. Pre-meal values were significantly higher in chow-AL-fed rats compared to the CR-fed groups after 1, 3, and 4 week of dietary treatment, with the exception of “BB” group at 4 weeks (one-way ANOVA plus Bonferroni's comparison for multiple testing, Supplementary Figure 2A); in contrast, no significant differences were observed among groups in post-meal glycemia values (Supplementary Figure 2B).

We then focused on the effect of these dietary treatments on the GM of CR-fed rats, according to the following considerations: (i) overfeeding by AL food consumption is a significant uncontrolled variable that might affect the total intake of bread provided with the diet; (ii) rather, rats fed with a CR regimen consume the whole feed before the following administration; (iii) the outcomes of the study are intended to be translated to normal-weight or lean individuals. To this aim, we collected stool, colon content, and liver samples from all experimental groups after 4 weeks of dietary exposure. Stool samples were subjected to both 16S rRNA gene sequencing and metaproteomic analysis, colonic contents to 16S rRNA gene sequencing only, and liver samples to host functional profile characterization only.

A total of 554,155 reads were obtained from fecal samples (50,378 on average per sample), and 469,049 reads from colon contents (39,087 on average per sample), corresponding to 130 microbial families and 160 microbial genera/species (Supplementary Dataset 1). In addition, a total of 96,913 microbial peptide-spectrum matches were obtained from fecal samples (8,810 on average per sample), corresponding to 1,850 microbial family-specific protein families and 1,882 microbial genus-specific protein families. Moreover, 32,416 (2,947 on average per sample) and 304,095 (25,341 on average per sample) host peptide-spectrum matches were identified from stool and liver samples, respectively, corresponding to 1,190 and 2,870 proteins (Supplementary Dataset 2). Differential analysis comparing host protein expression among dietary groups in stool and liver samples did not show any significant difference in abundance (data not shown).

Alpha and beta diversity among groups were evaluated considering genera/species, according to 16S rRNA gene sequencing (stool and colonic contents) and metaproteomic (stool only) data. No significant differences were observed for alpha diversity, according to Kruskal-Wallis test followed by Dunn's multiple comparison test on both Simpson and Shannon indexes (Supplementary Table 1); moreover, no significant clustering was detected based on PCoAs (PERMANOVA test, Supplementary Figure 3).

### Taxonomic Changes Induced by Sourdough Bread in the Rat Gut Microbiota

In order to study if differences in leavening (i.e., sourdough or baker's yeast) could affect the structure of the rat GM, we compared its taxonomic composition, at family and genus/species level, in BB and SB-fed rats, based on both 16S rRNA gene sequencing (colon and feces) and metaproteomic data.

**Figure 1:**
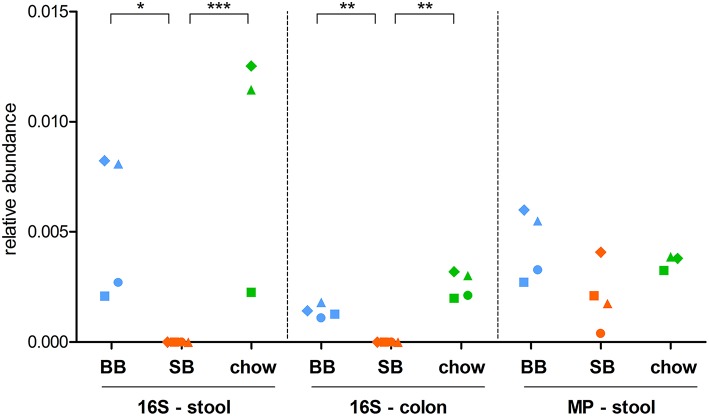
Relative abundance of the genus *Mycoplasma* in stool and colon content samples. Each dot represents a different rat, with dots with same shape and color being referred to the same rat. For stool samples, both 16S rRNA gene sequencing (16S) and metaproteomic (MP) data are shown. BB, rats fed chow supplemented with baker's yeast leavened bread (light blue); SB, rats fed chow supplemented with sourdough leavened bread (orange); chow, rats fed chow only (green). Statistically significant differences between groups (according to edgeR test followed by SGoF adjustment) are indicated with asterisks (^*^ = adjusted *p*-value < 0.05; ^**^ = adjusted *p*-value < 0.01; ^***^ = adjusted *p*-value < 0.00001).

Considering 16S rRNA gene sequencing, we found 11 and 4 taxa significantly changed in colon and feces, respectively (Table 1). Interestingly, the genus *Mycoplasma* and its corresponding family *Mycoplasmataceae* were consistently detected as more abundant in both the colonic and fecal microbiota of “BB” animals (Figure 1). Furthermore, the family *Cytophagaceae* was found as significantly more abundant in BB-fed rat stool, while the species *Alistipes indistinctus* and *Mucispirillum schaedleri* were significantly enriched in the colonic contents of BB-fed rats. On the other hand, the SB-associated GM was observed as enriched in 7 families, including *Verrucomicrobiaceae* in stool, and *Bacillaceae, DHVEG-1* (belonging to archeaea), *Moraxellaceae, Nitrospinaceae*, and *Thermaceae* in the colonic content.

**Table 1:** Differential taxa in gut microbiota of rats fed chow supplemented with bread leavened with baker's yeast (BB) vs. sourdough (SB).

**Taxon**	**16S-stool**		**16S-colon**		**MP-stool**	
	**logFC SB/BB**	**Adjusted *p*-value**	**logFC SB/BB**	**Adjusted *p*-value**	**logFC SB/BB**	**Adjusted *p*-value**
*A4b*			7.03	2.25 × 10^−3^		
*Bacillaceae*			6.65	6.58 × 10^−5^		
*Cytophagaceae*	−4.18	6.86 × 10^−3^				
*Desulfovibrionaceae*					−1.85	2.25 × 10^−3^
*DHVEG-1*			6.54	7.42 × 10^−4^		
*Lachnospiraceae*					−1.10	1.66 × 10^−2^
*Moraxellaceae*			7.04	1.78 × 10^−5^		
*Mycoplasmataceae*	−9.40	8.18 × 10^−4^	−7.33	4.53 × 10^−6^		
*Nitrospinaceae*			6.23	6.35 × 10^−3^		
*Thermaceae*			6.46	2.28 × 10^−4^		
*Verrucomicrobiaceae*	7.66	2.46 × 10^−3^				
*Alistipes indistinctus*	−5.16	3.81 × 10^−3^				
*Dubosiella*	3.57	2.33 × 10^−2^				
*Mucispirillum schaedleri*	−5.25	1.39 × 10^−3^				
*Mycoplasma*	−9.19	1.19 × 10^−2^	−7.95	4.80 × 10^−4^		
*YRC22*	−4.55	9.77 × 10^−3^				

*Families and genera/species with statistically significant differential abundance between BB- and SB-fed rats are listed. For each taxon, the adjusted p-value (edgeR test followed by SGoF adjustment) and the logarithm of the fold change (logFC), according to 16S rRNA gene sequencing (16S; stool and colonic contents) and metaproteomic (MP; stool only) data, are reported. Features are ordered based on alphabetical order, and families (up) are separated from genera/species (down)*.

When metaproteomic data were considered, 3 taxa exhibited a differential abundance between the two groups: *Lachnospiraceae* and *Desulfovibrionaceae*, enriched in BB-fed rats, and *Dubosiella* spp., more abundant in SB-fed rats (Table 1).

### Functional Changes Induced by Sourdough Bread in the Rat Gut Metaproteome

**Figure 2:**
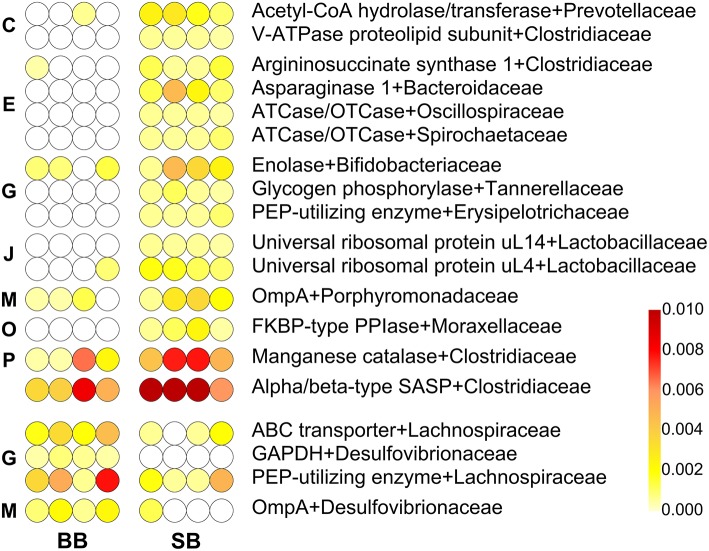
Differential family-specific microbial functions in rats fed chow supplemented with bread leavened with baker's yeast (BB) vs. sourdough (SB). In each line, a dot represents a single animal, with its color intensity being proportional to the relative abundance of that given microbial protein in that subject, according to the scale depicted in the bottom-right corner. Missing values (function not identified in that animal) are in white; features with missing values in the most abundant group were filtered out. The upper part of the heatmap lists functions with higher abundance in the fecal microbiota of SB-fed animals, while the lower part lists those with higher abundance in the fecal microbiota of BB-fed animals. Functions are ordered based on the Cluster of Orthologous Groups (COG) category to which they belong (C, Energy production and conversion; E, Amino acid transport and metabolism; G, Carbohydrate transport and metabolism; J, Translation, ribosomal structure and biogenesis; M, Cell wall/membrane/envelop biogenesis; O, Posttranslational modification, protein turnover, chaperones; P, Inorganic ion transport and metabolism), and then in alphabetical order.

In addition to the taxonomic composition, we also investigated the effect of the type of leavening agent on the GM activities; hence, we focused on differences in the expression of protein functions by comparing BB- and SB-fed rats. Protein sequences were first functionally annotated and then the functional information was combined with the taxonomic classification at family and genus levels. A total of 19 and 17 differential family-(Figure 2) and genus-specific (Supplementary Figure 4) functions were identified, respectively.

Among these, two *Desulfovibrio*-assigned functions, namely glyceraldehyde-3-phosphate dehydrogenase (GAPDH) and porin-like integral membrane protein (OmpA) families, and two *Lachnospiraceae*-assigned functions, phosphoenolpyruvate (PEP)-utilizing enzyme and ABC transporter superfamily, were detected as more abundant in BB-fed rats, in line with the taxonomic results based on metaproteomics (Taxonomic Changes Induced by Sourdough Bread in the Rat Gut Microbiota). In addition, bacterial ribosomal protein bS6 family, from *Clostridium*, and the *Lactobacillus*-specific glycosyl hydrolase 36 family, that includes α-galactosidase and α-N-acetylgalactosaminidase activities, showed the same behavior.

Interestingly, among the taxonomy-function combinations that exhibited a significant increase in the GM of SB-fed rats, we detected several protein families belonging to the amino acid metabolism and transport Cluster of Orthologous Groups (COG) category: aspartate and ornithine carbamoyltransferase (from *Oscillibacter* and *Treponema*), asparaginase 1 (*Bacteroides*), and type 1 argininosuccinate synthase (*Clostridiaceae*). Furthermore, we identified functions involved in energy production (*Prevotella*-specific Acetyl-CoA hydrolase/transferase and *Clostridium*-specific V-ATPase proteolipid subunit), translation (universal ribosomal protein uL4 and uL14, both assigned to *Lactobacillus*), and post-translational modification [FKBP-type peptidyl-prolyl cis-trans isomerase (PPIase) from *Acinetobacter*]. With reference to *Clostridium*, we also found manganese catalase and alpha/beta-type SASP, namely small acid-soluble spore protein; the latter binds the spore DNA and was the most abundant among the differential protein families. Finally, PEP-utilizing enzyme and OmpA presented an opposite differential trend depending on the specific taxonomic assignment, a possible effect of GM taxonomic variations due to SB- or BB-based diets. Thus, PEP-utilizing enzyme molecules assigned to *Dubosiella* were more abundant in SB-fed rats, whereas those assigned to *Lachnospiraceae* were more abundant in BB-fed rats; on the other hand, *Parabacteroides*-specific OmpA was more abundant in SB-fed rats, whereas *Desulfovibrio*-specific OmpA was more abundant in BB-fed rats.

### Taxonomic Changes Induced by *Carasau* Bread in the Rat Gut Microbiota Compared to Standard Chow Diet

To investigate if the supplementation with *carasau* bread to the common chow diet could significantly change the GM structure, we performed two separate differential analyses, i.e., “SB” vs. “chow” and “BB” vs. “chow” based on 16S rRNA gene sequencing (colon and feces) and metaproteomic data. When comparing “SB” and “chow” groups we identified 10 (stool 16S rRNA gene sequencing data), 9 (colon 16S rRNA gene sequencing data), and 11 (metaproteomics) taxa with an abundance significantly different between the two groups (Table 2). Most of these differences in 16S rRNA gene sequencing results were also found between “SB” and “BB” (Taxonomic Changes Induced by Sourdough Bread in the Rat Gut Microbiota). Indeed, in SB-fed rats a reduction of *Mycoplasmataceae* and its related genus *Mycoplasma* was again seen in both stool and colon (Figure 1), as well as a decrease of *Cytophagaceae* in stool, whereas *Verrucomicrobiaceae* (stool), *Bacillaceae, DHVEG-1, Moraxellaceae*, and *Thermaceae* (colon) were increased. Moreover, we found the GM of chow-fed group enriched in *Planococcaceae* and *Solibacillus*, both in stool and colon, while *Paraprevotella* was increased only in colon. Additionally, *Bradyrhizobium* and its related family *Bradyrhizobiaceae* were more abundant in SB-fed rat stool.

**Table 2:** Differential taxa in gut microbiota of rats fed chow supplemented with bread leavened with sourdough (SB) vs. unsupplemented (chow).

**Taxon**	**16S-stool**		**16S-colon**		**MP-stool**	
	**logFC SB/chow**	**Adjusted *p*-value**	**logFC SB/chow**	**Adjusted *p*-value**	**logFC SB/chow**	**Adjusted *p*-value**
*A4b*			5.49	2.25 × 10^−2^		
*Acidaminococcaceae*					1.98	5.42 × 10^−3^
*Bacillaceae*			5.14	2.99 × 10^−3^		
*Bradyrhizobiaceae*	5.06	3.17 × 10^−3^				
*Cytophagaceae*	−3.58	8.61 × 10^−3^				
*DHVEG-1*			5.46	8.18 × 10^−5^		
*Moraxellaceae*			5.41	8.54 × 10^−3^		
*Mycoplasmataceae*	−10.18	2.92 × 10^−5^	−7.93	1.22 × 10^−6^		
*Nocardiopsaceae*					2.16	1.44 × 10^−2^
*Planococcaceae*	−7.44	1.04 × 10^−4^	−5.92	2.14 × 10^−5^		
*Propionibacteriaceae*					2.69	3.56 × 10^−2^
*Thermaceae*			4.73	9.68 × 10^−4^		
*Sulfolobaceae*	−4.49	2.17 × 10^−2^				
*Verrucomicrobiaceae*	7.34	3.47 × 10^−4^				
*Bradyrhizobium*	5.07	2.79 × 10^−3^				
*Desulfococcus*					−3.69	1.20 × 10^−3^
*Desulfovibrio*					−2.04	1.99 × 10^−4^
*Dorea*					−1.74	6.07 × 10^−3^
*Ktedonobacter*					−2.19	4.86 × 10^−2^
*Marinactinospora*					2.26	9.90 × 10^−6^
*Metallosphaera*	−4.55	7.39 × 10^−3^				
*Mycoplasma*	−10.18	8.24 × 10^−6^	−8.31	5.94 × 10^−3^		
*Paraprevotella*			−9.52	1.51 × 10^−2^		
*Phascolarctobacterium*					1.95	4.99 × 10^−4^
*Pseudopropionibacterium*					2.77	7.62 × 10^−5^
*Solibacillus*	−7.51	2.97 × 10^−5^	−10.47	2.18 × 10^−3^		
*Turicibacter*					1.45	2.55 × 10^−2^

*Families and genera/species with statistically significant differential abundance between SB- and chow-fed rats are listed. For each taxon, the adjusted p-value (edgeR test followed by SGoF adjustment) and the logarithm of the fold change (logFC), according to 16S rRNA gene sequencing (16S; stool and colonic contents) and metaproteomic (MP; stool only) data, are reported. Features are ordered based on alphabetical order, and families (up) are separated from genera/species (down)*.

Metaproteomic analysis revealed an increased abundance of *Marinactinospora, Phascolarctobacterium, Pseudopropionibacterium*, and *Turicibacter* genera, as well as *Acidaminococcaceae* and *Propionibacteriaceae* families in the “SB” group; by contrast, *Desulfovibrio* and *Dorea* were significantly more abundant in chow-fed animals.

We also compared GMs from BB- and chow-fed rats, identifying 5 (stool) and 8 (colon) taxa as differentially abundant after 4 weeks of dietary treatment (Table 3). Interestingly, *Planococcaceae* and *Solibacillus* (both in stool and colon) and *Paraprevotella* (only in colon) were again enriched in chow-fed rats. Further, *Corynebacterium stationis* was enriched in feces of “chow” group. In contrast, *Phascolarctobacterium* was lower both in stool and colon of chow-fed rats (consistent with SB vs. chow metaproteomic data), as well as *[Ruminococcus]* in feces and *Ruminococcus bromii* in the colonic contents.

**Table 3:** Differential taxa in gut microbiota of rats fed chow supplemented with bread leavened with baker's yeast (BB) vs. unsupplemented (chow).

**Taxon**	**16S-stool**		**16S-colon**		**MP-stool**	
	**logFC BB/chow**	**Adjusted *p*-value**	**logFC BB/chow**	**Adjusted *p*-value**	**logFC BB/chow**	**Adjusted *p*-value**
*Flavobacteriaceae*			−4.98	4.52 × 10^−2^		
*Planococcaceae*	−9.60	3.56 × 10^−2^	−10.54	7.41 × 10^−3^		
*[Ruminococcus]*	4.19	2.64 × 10^−2^				
*Acetobacter*			5.07	1.27 × 10^−2^		
*Corynebacterium stationis*	−5.27	4.42 × 10^−3^				
*Paraprevotella*			−8.20	7.12 × 10^−5^		
*Phascolarctobacterium*	3.42	1.12 × 10^−2^	3.29	2.96 × 10^−2^		
*Ruminococcus bromii*			4.19	6.82 × 10^−4^		
*Solibacillus*	−11.48	1.87 × 10^−4^	−10.83	2.11 × 10^−5^		
*Veillonella dispar*			−4.40	5.12 × 10^−3^		

*Families and genera/species with statistically significant differential abundance between BB- and chow-fed rats are listed. For each taxon, the adjusted p-value (edgeR test followed by SGoF adjustment) and the logarithm of the fold change (logFC), according to 16S rRNA gene sequencing (16S; stool and colonic contents) and metaproteomic (MP; stool only) data, are reported. Features are ordered based on alphabetical order, and families (up) are separated from genera/species (down)*.

No taxonomic features were differentially represented in the GMs of BB- vs. chow-fed rats based on metaproteomic analysis.

### Functional Changes Induced by *Carasau* Bread in the Rat Gut Microbiota Compared to Standard Chow Diet

Finally, the taxa-specific functional changes occurring in the rat GM after diet supplementation with *carasau* bread were investigated. To this end we compared metaproteomic functions, based on their taxonomic annotation, of chow-fed rats with SB- and BB-fed rats.

**Figure 3:**
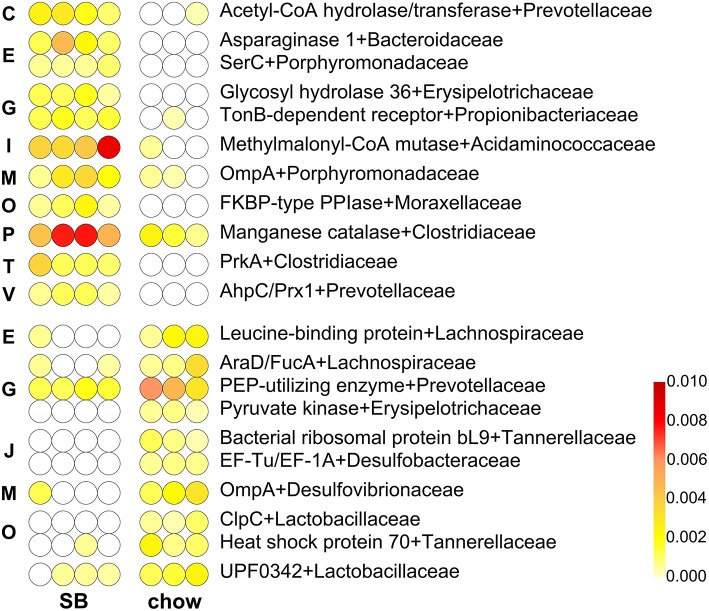
Differential family-specific microbial functions in rats fed chow supplemented with bread leavened with sourdough (SB) vs. chow only. In each line, a dot represents a single animal, with its color intensity being proportional to the relative abundance of that given microbial protein in that subject, according to the scale depicted in the bottom-right corner. Missing values (function not identified in that animal) are in white; features with missing values in the most abundant group were filtered out. The upper part of the heatmap lists functions with higher abundance in the fecal microbiota of SB-fed animals, while the lower part lists those with higher abundance in the fecal microbiota of chow-fed animals. Functions are ordered based on the Cluster of Orthologous Groups (COG) category to which they belong (C, Energy production and conversion; E, Amino acid transport and metabolism; G, Carbohydrate transport and metabolism; I, Lipid metabolism; J, Translation, ribosomal structure and biogenesis; M, Cell wall/membrane/envelop biogenesis; O, Posttranslational modification, protein turnover, chaperones; P, Inorganic ion transport and metabolism; T, Signal transduction mechanisms; V, Defense mechanisms), and then in alphabetical order.

A total of 21 family-(Figure 3) and 22 genus-specific functions (Supplementary Figure 5) were detected as differentially represented between “SB” and “chow” groups, while no differential features were observed between “BB” and “chow” groups. Not surprisingly, some of the differential functions more abundant in SB, i.e., acetyl-CoA hydrolase/transferase (assigned to *Prevotella*), asparaginase 1 (*Bacteroides*), FKBP-type PPIase (*Acinetobacter*), and manganese catalase (*Clostridium*), had been already found as more abundant in the “SB” group when compared with the “BB” group. Other SB-enriched features included functions implicated in amino acid metabolism and transport, namely serine-glycine hydroxymethyltransferase (SHMT) assigned to *Prevotella* and phosphoserine aminotransferase SerC assigned to *Porphyromonadaceae*. Finally, we found as differentially expressed also AhpC/Prx1 peroxiredoxin, (assigned to *Prevotella*), enolase (*Tannerella*), glycosyl hydrolase 36 (*Turicibacter*), methylmalonyl-CoA mutase (*Phascolarctobacterium*), serine-protein kinase PrkA (*Clostridium*), and TonB-dependent receptor (*Pseudopropionibacterium*).

Several functions involved in carbohydrate metabolism (L-ribulose-5-phosphate 4-epimerase/L-fuculose phosphate aldolase from *Lachnospiraceae*, PEP-utilizing enzyme from *Prevotella* and *Alloprevotella*, pyruvate kinase from *Turicibacter*, and transketolase family from *Prevotella*), translation (including bacterial ribosomal protein bL9 from *Parabacteroides* and the elongation factors EF-Ts, EF-Tu/EF-1A, EF-G/EF-2 from *Mediterranea, Desulfococcus*, and *Ruminococcus*, respectively), and post-translational modification (heat shock protein 70 assigned to *Parabacteroides* and ClpC protease from *Lactobacillaceae*) were enriched in “chow” group. Leucine-binding protein from *Lachnospiraceae* was also increased.

## Discussion

Sourdough bread is recognized to possess a great variety of valuable effects on nutrition and health. The worldwide interest in investigating its qualities with new and more robust methodologies is due to the existence of numerous traditional bakery products in many different countries, that are currently being re-discovered as relevant components of a well-balanced and “healthier” diet, as well as potentially useful as part of a therapeutic dietary intervention.

Indeed, compelling evidence was provided over the last few years to support nutrition and health claims of sourdough leavened bread. As extensively reviewed by Gobbetti et al., bread products obtained using LAB instead of baker's yeast are generally appreciated for the more complex and agreeable flavor and taste (Gobbetti et al., 2016). In addition, a lower glycemic index has been measured for bread leavened with LAB, when compared with the same type of bread leavened with *Saccharomyces cerevisiae* (Maioli et al., 2008; Poutanen et al., 2009; Stamataki et al., 2017). This feature, due to a lower amount of rapidly digestible starch in the small intestine, is accompanied by a larger amount of slowly digestible and resistant starch that reaches the colon, where it is degraded by colonic bacteria to produce short-chain fatty acids. The latter in turn provide energy to the colonic cells, reduce susceptibility to cancer, and control gut inflammation (van der Beek et al., 2017).

Hence, as different biochemical changes occur in sourdough and baker's yeast fermentations, both possibly impacting consumer's metabolism and health, the aim of our study was to compare their effects on the intestinal microbiota taxonomy and metabolism. We also aimed to evaluate for the first time the capability of metaproteomics to reveal the effects of these different bread making processes on GM structure and functions. Two kinds of flat bread (*carasau* bread) were obtained employing the same raw materials, manufacturing recipes, and processing conditions, but they were leavened with either yeast or sourdough fermentation.

In this study, microbiota taxonomy variation was assessed by means of 16S rRNA gene sequencing analysis. Diet supplementation with sourdough bread led to a reduction of specific members of the GM, belonging to genera as *Alistipes, Mucispirillum*, and *Mycoplasma*. Such changes might be due to differences in nutrients availability in LAB vs. baker's yeast fermented bread. Proteolysis occurring during lactic acid fermentation is expected to change the protein assortments and to reduce the amount of proteins reaching the colonic mucosa (Spicher and Nierle, 1988). Consequently, free amino acids, including alanine, glutamic acid, asparagine, and arginine are more abundant in sourdough, where the bacterial metabolic activities also increase the levels of dough acidity. This may explain the reduction of *Mucispirillum* that has been previously associated to protein-deficient diets (Navarro et al., 2018; Zhai et al., 2018). However, in this study, we did not measure the total proteins or free amino acids amounts in SB and BB. Consistently, *Alistipes* was also found associated to low protein diets and to the increase of dietary fatty acids (Agans et al., 2018; Kang et al., 2018; Wei et al., 2018). Interestingly, *Mycoplasma* is generally acknowledged as an intestinal pathobiont and it was associated with diet-induced obesity (Turnbaugh et al., 2008). Our data suggest the possibility that a low-fat diet, supplemented with SB, might reduce its growth. On the other hand, SB induced an increase of some bacterial taxa, including *Verrucomicrobiaceae*, in stool. These data are not consistent with those obtained by metaproteomics, where *Lachnospiraceae* and *Desulfovibrionaceae* were found enriched in the “BB” group, while *Dubosiella* spp. were significantly more abundant in the “SB” group. In this context, it should be noted that the use of a different database for protein assignment and the differences in the depth of coverage, with the metagenome enabling more complete coverage than the metaproteome, can result in different taxonomic annotations. For example, *Dubosiella* spp. is not listed in the used metagenomic database, being a recently proposed novel genus (Cox et al., 2017).

Our study of the rat metaproteome provides unique and important insights into the variations of gut microbial taxa, their proteins, and their functions associated with CR low-fat diet supplemented with BB or SB. A very intriguing observation is the higher abundance of asparaginases expressed by *Bacteroides* in SB-fed rats. Normally, bacteria control their catabolic enzyme synthesis and turnover according to the abundance of the relative substrates in the environment. Hence, the differential amount of asparaginase leads to hypothesize that higher amounts of asparagine reach the colonic mucosa of SB-fed rats compared to BB-fed rats. Asparagine and, to a lesser extent, other free amino acids have been reported to represent major precursors of acrylamide in baked bread (Tareke et al., 2002). Acrylamide is a known carcinogenic agent in rodents and a probable carcinogen in human. Although most of the dietary intake of acrylamide derives from fried potatoes and coffee, attention is also directed to baked bread since it might represent a significant source of this molecule. Notably, when comparing breads obtained with different leavening processes, higher acidity reduces the acrylamide formation in sourdough vs. yeast-fermented bread, despite the higher asparagine content in the former (Nasiri Esfahani et al., 2017). Our data are in keeping with those of Bartkiene et al., that have recently demonstrated the possibility to reduce acrylamide content down to 67.2% in sourdough bread prepared with selected LAB (Bartkiene et al., 2017). Since we could not find evidence of differential variation in relative abundances of *Bacteroides*, the increased amount of asparaginase in SB-fed GM points toward a “turning on” of *Bacteroides* asparaginase as inducible enzyme (Boeck et al., 1970). Asparaginase substrate, asparagine, is converted to aspartate and ammonium, in a metabolic pathway that provides carbon and nitrogen as components of many biomolecules. Asparagine catabolism in bacteria is therefore important to compete against other bacterial members of the GM community and it is a significant virulence determinant for many enteric pathogens (Scotti et al., 2010; McLaughlin et al., 2017). At the same time, bacterial competition for asparagine, and its degradation, might be beneficial for the host, reducing the risk of colon cancer and/or its progression. Asparaginase is indeed well known as key in the treatment of acute lymphoblastic leukemia and its potential as “anti-colon cancer protein” has been recently proposed (El-Naggar et al., 2016; Miyo et al., 2016; Toda et al., 2016).

Other enzymes might be regulated in response to specific metabolite(s) produced or enhanced by SB or BB, but their limit of detection could be below that of our metaproteomic approach. Or else, a number of these enzymes might be regulated by allosteric mechanism, rather than by a change in their expression level.

Another group of five protein families, expressed by *Clostridium*, were observed to change their abundance in animals fed SB-supplemented diet. Of these, the bacterial ribosomal protein bS6 showed higher relative abundance in BB-fed rats. On the contrary, manganese catalase, small acid-soluble proteins (SASP), Ser/Thr kinase PrkA, and V-ATPase proteolipid subunit were higher in the GM of SB-fed rats. These proteins have all been reported to be involved in *Clostridium* sporulation. SASPs, in particular, play an important role in protecting DNA against damage by heat, UV radiation, or enzymic degradation in dormant bacterial endospores (Wetzel and Fischer, 2015). ATP synthase subunits were shown to be upregulated during late sporulation, possibly to meet energy demands, both in *Bacillus* and in *Clostridium* (Wang et al., 2013; Liu et al., 2016). Manganese catalase is a spore coat protein with an important role of H_2_O_2_ detoxification (Permpoonpattana et al., 2013). Finally, also PrkA is involved in sporulation, although its role is not yet well understood (Pompeo et al., 2016). Taken together, these data strongly suggest that consumption of sourdough might increase the subset of metabolically dormant *Clostridium* spp. in the GM. Among *Clostridium* spp., the most studied one is the gut pathobiont *C. difficile*, the causative agent of pseudomembranous colitis and toxic megacolon, whose pathogenicity is potentiated by antibiotic treatment. In fact, a healthy microbiota is expected to dampen *C. difficile* germination, probably through *C. difficile*-growth-inhibitory secondary bile acids (Shen, 2015). To this end, the GM of SB-fed rats might have a specific effect on *Clostridium* sporulation.

In conclusion, we provide evidence that consumption of sourdough-leavened bread has the potential to significantly change the taxonomy of the GM and the metabolic functions of some of its most important members, including *Bacteroides* and *Clostridium*. Further, the results of this study confirm that metaproteomics is able to pinpoint the impact of food processing technologies on microbial enzymes and related metabolites, which are in turn able to reach the gut mucosa and exert their potential effect on the consumer's health at intestinal and systemic level.

## Data Availability

Raw read sequences were deposited in the European Nucleotide Archive under the Project Accession Number PRJEB29264.

The mass spectrometry proteomics data have been deposited to the ProteomeXchange Consortium via the PRIDE (Vizcaino et al., 2016) partner repository with the dataset identifier PXD011441.

## Ethics Statement

Animal studies were reviewed and approved by the Institutional Animal Care and Use Committee of the University of Cagliari and were performed in accordance with the relevant guidelines and regulations (authorization of the Italian Health Ministry No. 840/2016-PR).

## Author Contributions

MA performed 16S rRNA gene sequencing sample preparation and analysis, contributed to data interpretation, and wrote the manuscript. AP performed metaproteomics sample preparation, mass spectrometry analysis, and contributed to data interpretation and to critically revise the manuscript. AT performed metaproteomics sample preparation, supervised the global data analysis and interpretation, and contributed to critically revise the manuscript. CF performed 16S rRNA gene sequencing sample preparation and analysis. DP supervised mass spectrometry analysis. MS and FM performed animal experiments and sample collection. EL conceived and coordinated the study. SU conceived and coordinated the study, contributed to data interpretation, and wrote the manuscript. All authors read and approved the final version of the manuscript.

### Conflict of Interest Statement

The authors declare that the research was conducted in the absence of any commercial or financial relationships that could be construed as a potential conflict of interest.
