# Supplementary material for: Fecal Metaproteomic Analysis Reveals Unique Changes of the Gut Microbiome Functions After Consumption of Sourdough Carasau Bread
Source: Front Microbiol. 2019 Jul 30;10:1733. doi: 10.3389/fmicb.2019.01733 (PMC6682701; doi:10.3389/fmicb.2019.01733)
Supplement: Supplementary file 1 [file Data_Sheet_1.docx]

Supplementary Material

# Supplementary Data

**Supplementary Dataset S1. Tables reporting general assignments, taxonomic annotations and differential features concerning 16S rRNA gene sequencing results.**

**Supplementary Dataset S2. Tables reporting general identifications, taxonomic and functional annotations and differential features concerning (meta)proteomic results.**

# Supplementary Figures and Tables

## Supplementary Figures

**
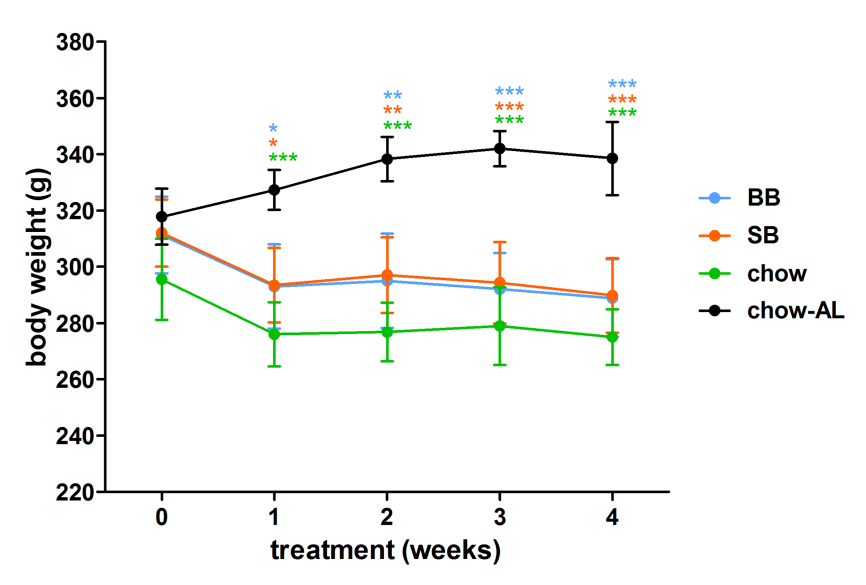
**

**Supplementary Figure S1. Body weight curves.** Body weight values measured in rats during the diet treatment. Error bars indicate standard deviation. BB, rats fed chow supplemented with baker's yeast leavened bread (light blue); SB, rats fed chow supplemented with sourdough leavened bread (orange); chow, rats fed chow only (green); chow-AL, rats fed *ad libitum* with chow only (black). Asterisks near black dots indicate a statistically significant difference (according to one-way ANOVA followed by Bonferroni's multiple comparison test) between chow-AL and a group marked with the corresponding color (e.g. orange asterisk indicates a significant difference between chow-AL and SB). * = adjusted p-value < 0.05; ** = adjusted p-value < 0.01; *** = adjusted p-value < 0.001.

**
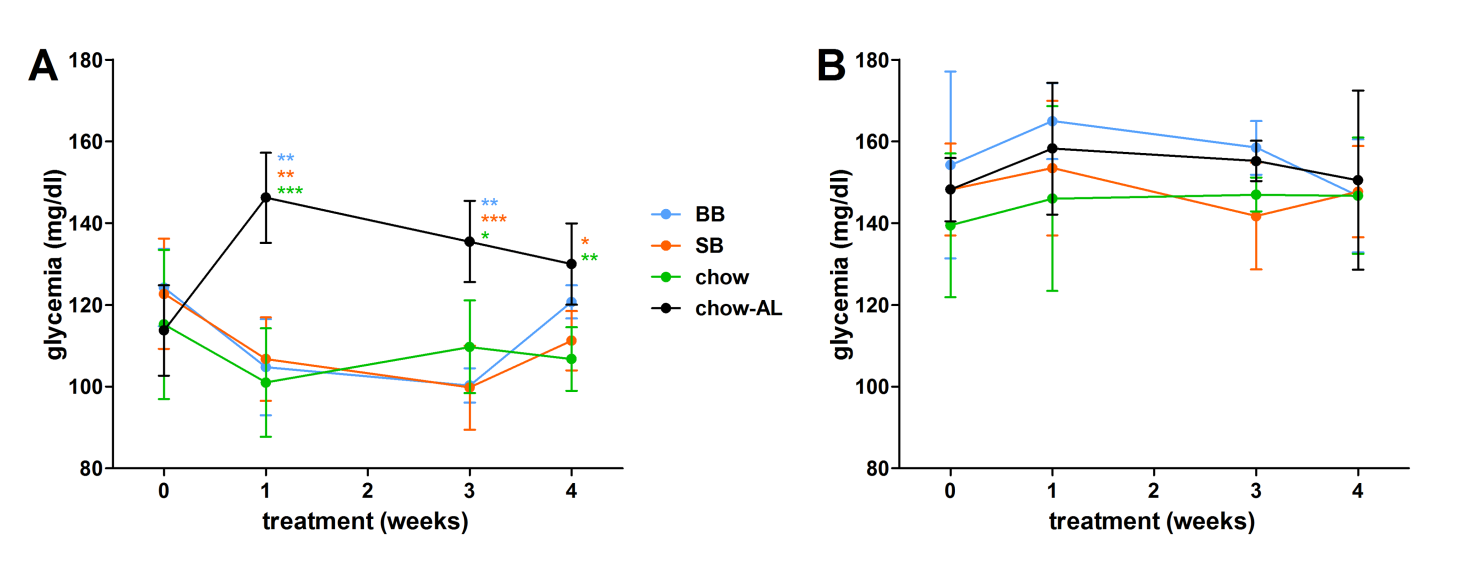
**

**Supplementary Figure S2. Glycemia curves.** (A) Glycemia values measured in rats one hour before feeding. (B) Glycemia values measured in rats two hours after feeding. Error bars indicate standard deviation. BB, rats fed chow supplemented with baker's yeast leavened bread (light blue); SB, rats fed chow supplemented with sourdough leavened bread (orange); chow, rats fed chow only (green); chow-AL, rats fed *ad libitum* with chow only (black). Asterisks near black dots indicate a statistically significant difference (according to one-way ANOVA followed by Bonferroni's multiple comparison test) between chow-AL and a group marked with the corresponding color (e.g. orange asterisk indicates a significant difference between chow-AL and SB). * = adjusted p-value < 0.05; ** = adjusted p-value < 0.01; *** = adjusted p-value < 0.001.


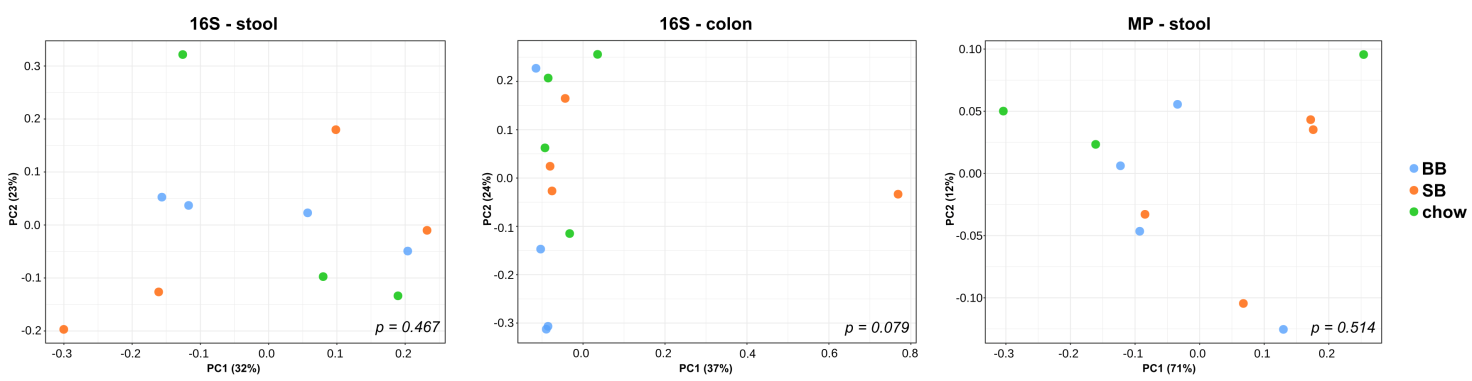


**Supplementary Figure S3. Beta diversity among gut microbiota of rats fed chow supplemented or not with *carasau* bread.** BB, rats fed chow supplemented with baker's yeast leavened bread (light blue); SB, rats fed chow supplemented with sourdough leavened bread (orange); chow, rats fed chow only (green). Principal coordinate analyses (PCoA) were carried out according to 16S rRNA gene sequencing (16S; stool and colonic contents) and metaproteomic (MP; stool only) taxonomic data at the genus level. For each PCoA, permutational multivariate analysis of variance (PERMANOVA) p-value is also reported.

**
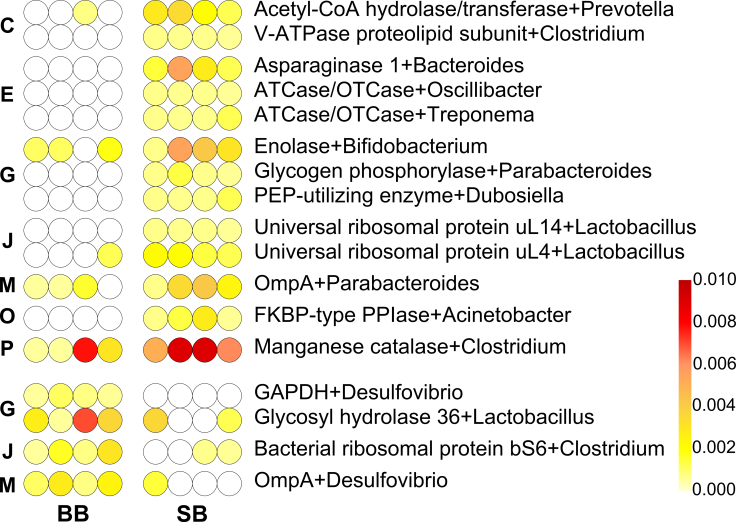
**

**Supplementary Figure S4. Differential genus-specific microbial functions in rats fed chow supplemented with bread leavened with baker's yeast (BB) *vs* sourdough (SB).** In each line, a dot represents a single animal, with its color intensity being proportional to the relative abundance of that given microbial protein in that subject, according to the scale depicted in the bottom-right corner. Missing values (function not identified in that animal) are in white; features with missing values in the most abundant group were filtered out. The upper part of the heatmap lists functions with higher abundance in the fecal microbiota of SB-fed animals, while the lower part lists those with higher abundance in the fecal microbiota of BB-fed animals. Functions are ordered based on the Cluster of Orthologous Groups (COG) category to which they belong (C, Energy production and conversion; E, Amino acid transport and metabolism; G, Carbohydrate transport and metabolism; J, Translation, ribosomal structure and biogenesis; M, Cell wall/membrane/envelop biogenesis; O, Posttranslational modification, protein turnover, chaperones; P, Inorganic ion transport and metabolism), and then in alphabetical order.

**
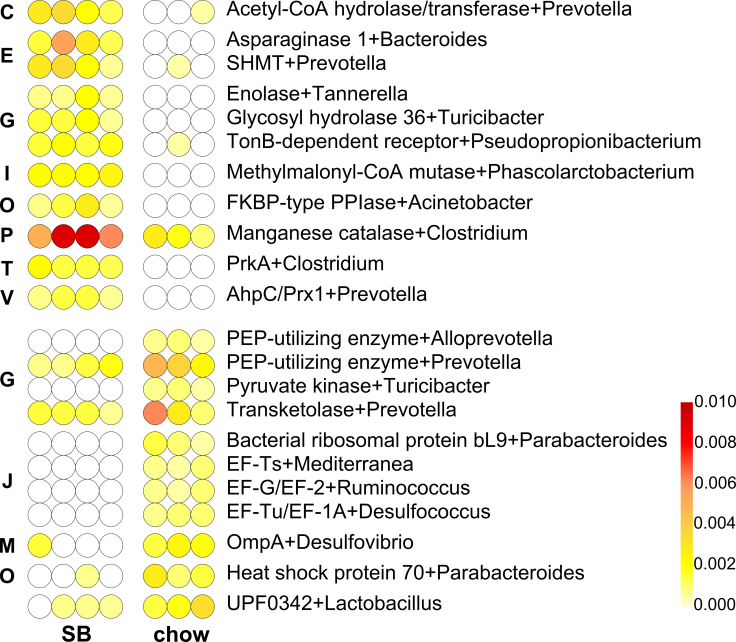
**

**Supplementary Figure S5. Differential genus-specific microbial functions in rats fed chow supplemented with bread leavened with sourdough (SB) *vs* chow only.** In each line, a dot represents a single animal, with its color intensity being proportional to the relative abundance of that given microbial protein in that subject, according to the scale depicted in the bottom-right corner. Missing values (function not identified in that animal) are in white; features with missing values in the most abundant group were filtered out. The upper part of the heatmap lists functions with higher abundance in the fecal microbiota of SB-fed animals, while the lower part lists those with higher abundance in the fecal microbiota of chow-fed animals. Functions are ordered based on the Cluster of Orthologous Groups (COG) category to which they belong (C, Energy production and conversion; E, Amino acid transport and metabolism; G, Carbohydrate transport and metabolism; I, Lipid metabolism; J, Translation, ribosomal structure and biogenesis; M, Cell wall/membrane/envelop biogenesis; O, Posttranslational modification, protein turnover, chaperones; P, Inorganic ion transport and metabolism; T, Signal transduction mechanisms; V, Defense mechanisms), and then in alphabetical order.

## Supplementary Tables

**Supplementary Table S1. Alpha diversity within gut microbiota of rats fed chow supplemented or not with *carasau* bread.** Simpson and Shannon index values calculated at genus/species level and their respective standard deviations are showed. 16S, 16S rRNA gene sequencing; MP, metaproteomic; BB, rats fed chow supplemented with baker's yeast leavened bread; SB, rats fed chow supplemented with sourdough leavened bread; chow, rats fed chow only. No statistically significant differences between groups (according to Kruskal-Wallis test followed by Dunn's multiple comparison test) were found.

| **Sample group** | **16S - stool** | | **16S - colon** | | **MP - stool** | |
| --- | --- | --- | --- | --- | --- | --- |
|  | **Simpson** | **Shannon** | **Simpson** | **Shannon** | **Simpson** | **Shannon** |
| BB | 0.866 ± 0.058 | 2.650 ± 0.211 | 0.859 ± 0.033 | 2.514 ± 0.187 | 0.913 ± 0.021 | 3.116 ± 0.193 |
| SB | 0.851 ± 0.049 | 2.456 ± 0.256 | 0.669 ± 0.335 | 1.855 ± 0.967 | 0.889 ± 0.023 | 2.914 ± 0.173 |
| chow | 0.881 ± 0.014 | 2.640 ± 0.079 | 0.809 ± 0.108 | 2.388 ± 0.357 | 0.896 ± 0.055 | 2.998 ± 0.363 |
